# Clinical features of outpatients with somatization symptoms treated at a Japanese psychosomatic medicine clinic

**DOI:** 10.1186/s13030-017-0104-x

**Published:** 2017-06-28

**Authors:** Yuzo Nakamura, Takeaki Takeuchi, Kazuaki Hashimoto, Masahiro Hashizume

**Affiliations:** Department of Psychosomatic Medicine, Toho University School of Medicine, 6-11-1 Omori-Nishi, Ota-ku, Tokyo, 143-8541 Japan

**Keywords:** Functional somatic symptoms (FSS), Medically unexplained symptoms (MUS), Somatization

## Abstract

**Background:**

Somatization is produced due to the summation of psychological factors, irrespective of the presence or absence of physical factors. A group of diseases with severe pain and other disorders exhibit so-called Medically Unexplained Symptoms (MUS), and the characteristics of patients with MUS are largely unexplained. In this paper, the characteristics of a series of new patients with somatization treated in a Japanese university hospital are discussed.

**Method:**

The subjects were 871 patients who newly visited the Department of Psychosomatic Medicine, Toho University Omori Medical Center between January and December of 2015. Under the assumption that the definition of somatization is same as that of MUS, the correlation between somatization and the age, sex, academic background, chief complaints, reasons for visiting the medical center, diagnosis, symptoms, presence or absence of a referral form, continued treatment after the first visit, and marital status of these patients at the time of their respective examinations were evaluated.

**Results:**

Of the patients studied, 68% suffered from somatization. Among them, 11% met the definition of Functional Somatic Symptoms (FSS) and 74% had somatization associated with mood disorder or anxiety disorder. Digestive symptoms were reported by 33%, headaches by 24%, and unusual sensations by 21%. Whereas no correlation was found between somatization symptoms and the patients’ academic background, marital history, or medical history after the first visit, a positive correlation (*p* < 0.05) was found between somatization and patients who had been referred by their doctor.

**Conclusion:**

Many of the studied patients who suffered from somatization, regardless of age and sex, were referred to us by doctors from other hospitals. It was concluded that many patients difficult to diagnose or deal with are referred the Department of Psychosomatic Medicine of Japanese university hospitals, thus these hospitals must assume great responsibility for preventing mistaken diagnoses by conducting effective psychological treatment and thorough medical examinations.

## Background

Somatization is a syndrome requiring medical assistance that is caused by psychological factors or the summation of psychological factors, regardless of the presence or absence of physical factors [1]. Such somatization includes somatoform disorders and functional somatic symptoms as well as somatization as a defense mechanism. In the 1990’s, a group of diseases that showed lasting physical symptoms and presented severe pain and serious disorders in comparison with those for a medically accountable clinical condition were identified and are called Medically Unexplained Symptoms (MUS). In 1999, Barsky defined the symptoms of this group of diseases as “Functional Somatic Symptoms (FSS)” [2]. In the actual clinical setting, however, there exist patients who do not fit the definitions of somatoform disorders and FSS. For example, the prognosis of mood and anxiety disorders along with a somatization symptom is thought to be largely related to the presence or absence of such a somatization symptom [3, 4].

Patients with a somatization symptom tend to need medical assistance. The psychological problems of such patients are frequently not addressed because the patients are seen by doctors with other areas of expertise, which results in increased medical expenses [5–8]. This situation can be attributed to problems on both the medical professional and patient sides. Complaints by patients suffering from a somatization symptom range from those more relating to the body than to those more relating to the mind and “cannot be well explained as the result of physical diseases and drugs”. A problem of the medical professional is that, because such complaints are difficult for them, the complaints are handled only as a matter of physical disease, with the implementation of repetitive medical examinations instead of providing psychological care. Meanwhile, patient problem lies in the fact that many do doctor shopping to avoid psychological intervention. Patients with a somatization symptom need to be treated with a mind/body approach [9–11]. While the origin of psychosomatic medicine dates back to the days of Hippocrates, who is said to be the founder of medicine, the basics of current psychosomatic medicine are believed to be from the psychoanalysis and dynamic psychiatry of the 1930’s [12]. In Japan, psychosomatic medicine presently targets a patient group that overlaps that of general medicine that advocates holistic medicine, but it rather provides support in terms of behavioral and preventive medicine and offers psychological help to those with FSS or MUS [13, 14].

In this paper, the characteristics of a series of new patients with a somatization symptom treated in a Japanese university hospital are examined. Ten patients diagnosed as having an organic disease after referral stating that they were suffering from a somatization symptom are also highlighted.

## Subjects and methods

### Participants

This study targeted 871 patients (374 male and 497 female) who paid their first visit to the Division of Psychosomatic Medicine, Toho University Omori Medical Center between January 4 and December 28, 2015.

### Definition of somatization and other variables

**Fig. 1:**
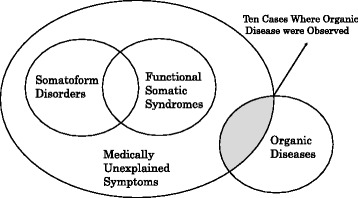
Definition of medically unexplained symptom’s (somatization) used in our study. Newly made based on reference eight

The definition of a somatization symptom used in this paper that the patient was suffering from MUS, as in Fig. 1. According to the presence or absence of a somatization symptom (dichotomous data), data were collected on the patient’s age, sex, academic background (a college graduate or not), major complaints, reasons for visiting the medical center (“Patient initiative” or “Referral by a Doctor”), diagnosis, symptoms (insomnia, general physical malaise, headaches, general cardiovascular malaise, general respiratory malaise, general digestive malaise, dizziness, and unusual sensations), the presence or absence of a referral form, continued treatment after the first visit (the presence of absence of continued hospital visits), and marital status. Patients with psychiatric symptoms alone were excluded because our department does not cover them. Anxiety and depression were diagnosed through an interview by an experienced physician in accordance with the diagnostic criteria of DSM - 5.

### Statistical analysis

We first evaluated the patient characteristics in a descriptive manner as somatization or no somatization. Logistic regression analysis adjusted by age and sex was conducted to test possible correlations between the patients with a somatization symptom and ①their academic background, ②reason for visit, ③the presence of absence of a referral form, ④marital status, and ⑤ continued treatment after the first visit. The characteristics of ten patients with a diagnosis of an organic disease after referral for a somatization symptom are described. We performed all analyses with the software program SPSS version 18.0 (SPSS Inc., Chicago, IL, USA). This research was approved by the Ethics Committee of Toho University Omori Medical Center (No. M-16268).

## Results

The basic attributes of the patients of this study are listed in Table 1. Of them, 68.1% had a somatization symptom, and 66 of 606 patients (10.9%) fit the definition of FSS, most of whom had mood and/or anxiety disorders (73.5%). Of the manifested somatization symptoms, digestive symptoms were reported by 32.8% of the patients, headaches for 23.8%, and unusual sensations for 21.1%. General malaise in one or two body parts was a complaint of 60% or more of the patients with a somatization symptom.

**Table 1:** Comparison of Patient Characteristics by Presence or Absence of Somatization(*N* = 871)

	Somatization		
	Positive (*N* = 606)	Negative (*N* = 265)	*p* value
Age, years (SD)	43.6 (18.9)	42.6 (18.6)	0.44
Sex (%)			0.60
Male	264 (30.3)	110 (12.6)	
Female	342 (39.3)	155 (17.8)	
Academic history (%)			0.58
College or above	197 (22.6)	81 (9.3)	
High school or under	359 (41.2)	176 (20.2)	
Unknown	50 (5.7)	8 (1.0)	
Visit at patient’s initiative (%)			0.05
Positive	311 (51.3)	154 (58.1)	
Negative	294 (48.5)	109 (41.1)	
Unknown	1 (<0.1)	2 (<0.1)	
Referral form (%)			0.01
Positive	391 (44.9)	144 (16.5)	
Negative	215 (24.7)	121 (13.9)	
Marital history (%)			0.30
Positive	255 (29.3)	101 (11.6)	
Negative	258 (29.6)	114 (13.1)	
Unknown	93 (10.7)	50 (5.7)	
Continued treatment after first visit (%)^a^			0.16
Positive	376 (43.2)	125 (14.4)	
Negative	184 (21.1)	78 (9.0)	

^a^”Continued treatment after first visit” excludes cases unknown after the first visit. Two-tailed t-tests were used to compare the mean differences. For categorical variables, Chi-square tests or proportion tests were used

The correlations between the somatization of the studied patients and their basic attributes are shown in Table 2. No correlation was found between somatization and academic background, marital history, or medical history after the first visit, but a positive correlation (*p* < 0.05) was found between somatization and patients referred by their doctor. Somatization and patient initiated visit were marginally related (*p* = 0.05). In addition, three patients who had been referred as suffering from a somatization symptom were revealed to have an organic disease, diagnosed by a through examination by the doctors at the medical center (Table 3). Of the 871 patients, 10 (1.1%) were diagnosed as having an organic disease in 2015. Many of them had a somatization symptom similar to mild panic attacks with palpitations. They had been referred to the medical center because they had not shown improvement through treatment for their panic attacks. Electroencephalographic examination of three patients whose somatization symptoms were accompanied by disturbed consciousness showed that they had been experiencing partial seizures of psychogenic, non-organic epilepsy or epilepsy, the symptoms of which were improved by the prescription of an anti-epilepsy drug. Also, two patients who had been initially referred as suffering from intractable diarrhea type Irritable Bowel Syndrome (IBS) showed irregular symptoms, and consequently our medical center’s doctor specializing in gastrointestinal diseases conducted a close examination and diagnosed small intestinal bacterial overgrowth syndrome and/or bile acid resorption disorder, the symptoms of which were resolved by specific treatment for these diseases. Unfortunately, however, one patient died who suffered from pachymeningitis hypertrophica.

**Table 2:** Correlation between Patient Characteristics and Somatization (*N* = 871)

		Crude OR(95% CI)	*p* value	Adjusted OR^a^(95% CI)	*p* value
Academic history (*N* = 813)	College or above (reference)	1.00		1.00	
	High school or under	1.19(0.87–1.63)	0.27	1.17(0.85–1.63)	0.34
Visit at patient’s initiative (*N* = 868)	Negative (reference)	1.00		1.00	
	Positive	0.74(0.55–1.00)	0.05	0.75(0.56–1.00)	0.05
Referral form (*N* = 871)	Negative (reference)	1.00		1.00	
	Positive	1.47(1.09–1.97)	0.01	1.46(1.09–1.98)	0.01
Marital history (*N* = 728)	Negative (reference)	1.00		1.00	
	Positive	1.12(0.81–1.53)	0.50	1.20(0.79–1.82)	0.38
Continued treatmentafter first visit (*N* = 763)	Negative (reference)	1.00		1.00	
	Positive	1.28(0.91–1.78)	0.15	1.24(0.89–1.75)	0.20

^a^Adjusted by age and sex

**Table 3:** Characteristics of Ten Patients with a Diagnosis of Organic Disease after being Diagnosed with Somatization

Age	Sex	Chief Complaint	Final Diagnosis	Referral Form	Initial response
37	Female	Panic Attack	Psychogenic Non-organic Epilepsy	Negative	Referred to Another Hospital
29	Male	Palpitation	Epilepsy, Social Anxiety Disorder	Negative	Referred to Psychiatrist
24	Male	Loss of Consciousness	Epilepsy	Positive	Provided Continued Treatment
68	Female	Headache	Chronic Hypertrophic Pachymeningitis	Positive	Provided Continued Treatment
79	Female	Difficulty in Moving Legs	Parkinsonism	Positive	Returned to Previous Doctor
46	Female	Diarrhea, Bad Smell from Body	Intestinal Bacterial Overgrowth Syndrome	Negative	Provided Continued Treatment
32	Male	Diarrhea	Bile Acid Malabsorption	Positive	Provided Continued Treatment
78	Female	Extremity muscle weakness	Spinal Cord Tumor	Positive	Provided Continued Treatment
69	Male	Itchy Feeling in Legs	Restless Legs Syndrome	Positive	Provided Continued Treatment
73	Male	Tremulousness	Drug-induced Parkinsonism	Positive	Returned to Previous Doctor

## Discussion

In this study, most of the somatization symptoms were associated with mood and anxiety disorders, and approximately 10% of the patients had FSS. This phenomenon is the result of greater attention in primary care being given to MUSs and FSSs [15]. In addition, patients with somatization symptoms, which are considered difficult to handle in primal care, are increasingly referred to our department. Further, although other studies have reported that the most commonly complained about symptoms are dorsal and joint pains [16], this study shows that many somatization symptoms are a sign of somatic diseases, such as digestive diseases and headaches. The disparity seen in the studies may have been brought about by differences in culture and medical institutions. Therefore, it will be important in the future to conduct a similar, large-scale survey that covers other facilities and that considers cultural and generational characteristics.

Although a significant difference in age and academic history among those complaining about somatization symptoms has been reported [16], this study demonstrated significant correlations only for the presence or absence of a referral and the reason for the visit. This outcome reflects that many general internal medicine and general medicine clinics see patients who present reporting physical syndromes but who they diagnose as having syndromes attributable to psychological factors. They continue to treat these patients because few such patients are willingly to visit a medical center for the treatment of psychological disorders [17]. The outcome of this study supports the characteristics of patients visiting psychosomatic medicine hospitals in Japan that were reported in our previous studies [18, 19].

The most notable finding of this study is that there were ten patients who had actually suffered from an organic disease that caused a somatization symptom, even though their referral letter reported only that they had a somatization symptom. That epilepsy as an organic disease, as observed in this study, and mild panic attack are correlated has been previously reported [20]. It is possible to make a distinction between the two by closely checking the patient’s medical history. In addition, because the relevant patients had presented symptoms that were not typical of IBS, those referred as suffering from intractable diarrhea were able to receive a final diagnosis of small intestinal bacterial overgrowth syndrome [21] or bile acid malabsorption [22]. Pachymeningitis hypertrophica [23] is an intractable disease that would be difficult to identify in primary care or by doctors not familiar with this field. Further, of the ten patients who were revealed to have an organic disease, eight made complaints about two or more body parts, which may be responsible for the difficulty of diagnosis. Doctors in many countries are discussing how to deal with patients who have somatization symptoms [24]. In particular, doctors who do not specialize in psychological treatment have a tendency to think that patients suffering from a somatization symptom are “difficult” to deal with [25] and seem to think that it takes more energy to take care of such patients than regular patients. In fact, in this study eight patients who had a diagnosis of an organic disease complained about malaise and, accordingly, it took more effort to determine these patients’ major complaints than those of usual patients. The findings of this study show that many patients suffering from a somatization symptom do not want to be treated as a mental patient and therefore receive only primary care, which indicates the importance of designing systems for systematically caring for patients with a somatization symptom in the primal care setting.

Also, it has been reported that the somatization symptoms of aged patients might be affected by environmental determinants, including living alone, as a psychological factor [26, 27] but this study was not able to clarify this point.

Many of the patients suffering from somatization in this study, regardless of their age and sex, had been referred to us by doctors from other hospitals. We can conclude that the Department of Psychosomatic Medicine of Japanese university hospitals is the proper place to treat patients, regardless of age and sex, who have been difficult to diagnose or to handle and that they play a major role in preventing mistaken diagnoses by conducting intensive psychological treatment and providing detailed medical examinations.

## Conclusions

The characteristics of a series of patients newly treated for somatization in a Japanese university hospital were discussed. Most patients had somatization associated with mood disorder or anxiety disorder. A positive tendency was recognized between somatization and referral by a primary care physician. The psychosomatic Medicine Departments of Japanese university hospitals should assume the responsibility for preventing mistaken diagnosis by conducting intensive psychological treatment and providing detailed medical examinations.

## Abbreviations

FSS: Functional Somatic Symptoms
IBS: Irritable Bowel Syndrome
MUS: Medically unexplained symptoms
